# Genotypic and Pathotypic Characterization of Newcastle Disease Viruses from India

**DOI:** 10.1371/journal.pone.0028414

**Published:** 2011-12-09

**Authors:** Krishnaswamy G. Tirumurugaan, Sunil Kapgate, Manavalan K. Vinupriya, Kumanan Vijayarani, Kathaperumal Kumanan, Subbiah Elankumaran

**Affiliations:** 1 Department of Animal Biotechnology, Madras Veterinary College, Tamil Nadu Veterinary and Animal Sciences University, Chennai, India; 2 Department of Biomedical Sciences and Pathobiology, Virginia-Maryland Regional College of Veterinary Medicine, Virginia Polytechnic Institute and State University, Blacksburg, Virginia, United States of America; Nanyang Technological University, Singapore

## Abstract

Newcastle disease virus (NDV) is an avian paramyxovirus that causes significant economic losses to the poultry industry in most parts of the world. The susceptibility of a wide variety of avian species coupled with synanthropic bird reservoirs has contributed to the vast genomic diversity of this virus as well as diagnostic failures. Since the first panzootic in 1926, Newcastle disease (ND) became enzootic in India with recurrent outbreaks in multiple avian species. The genetic characteristics of circulating strains in India, however, are largely unknown. To understand the nature of NDV genotypes in India, we characterized two representative strains isolated 13 years apart from a chicken and a pigeon by complete genome sequence analysis and pathotyping. The viruses were characterized as velogenic by pathogenicity indices devised to distinguish these strains. The genome length was 15,186 nucleotides (nt) and consisted of six non-overlapping genes, with conserved and complementary 3′ leader and 5′ trailer regions, conserved gene starts, gene stops, and intergenic sequences similar to those in avian paramyxovirus 1 (APMV-1) strains. Matrix gene sequence analysis grouped the pigeon isolate with APMV-1 strains. Phylogeny based on the fusion (F), and hemagglutinin (HN) genes and complete genome sequence grouped these viruses into genotype IV. Genotype IV strains are considered to have “died out” after the first panzootic (1926–1960) of ND. But, our results suggest that there is persistence of genotype IV strains in India.

## Introduction

Newcastle disease (ND) is one of the most economically important and prevalent poultry diseases around the world. Newcastle disease virus (NDV), the prototype avian paramyxovirus serotype 1 (APMV-1), belongs to the genus Avulavirus, in the family *Paramyxoviridae* and is the causative agent of ND [1]. NDV has a negative sense, single stranded RNA genome of approximately 15 kb that contains six genes in the order of 3′-NP-P-M-F.HN-L-5′coding for the nucleocapsid protein (NP), phosphoprotein (P), matrix protein (M), fusion protein (F), an attachment protein, the haemagglutinin-neuraminidase (HN), and a large polymerase protein (L) [2]. Two additional proteins, V and W are derived from the P gene by a process called RNA editing [3].

The key contributor to APMV-1 pathogenicity is the formation of an active fusion protein upon cleavage of the F protein precursor (F_o_) as well as the presence of a number of basic residues in the fusion protein cleavage site (FPCS) [4], [5]. Although APMV-1 is considered to belong to single serotype, antigenic and genetic diversity have been recognized [6], [7], [9]. There are two different systems of classifying NDV genotypes based on the FPCS sequence with nominal discrepancies. The first system classifies NDV in to 6 lineages with 13 sub-lineages [7] and three additional sublineages were added later [8]. The second system divides NDV into class I (with 9 genotypes) and class II (with 11 genotypes) [7]. APMV-I strains have at least three genome lengths: 15,186, 15,192, and 15,198 nt [10]. The class I viruses with a genome size of 15,198-nt are distributed worldwide in wild birds and generally avirulent to chickens and have also been isolated from live bird market samples [7], [11]. The class II viruses include most virulent and some avirulent and vaccine viruses [10].

Class-II NDV of multiple genotypes has been shown to circulate worldwide [12]. The genotype I, II, III and IV strains are responsible for the first panzootic during 1920 to 1960s [13], while strains of genotype V and VI resulted in the second panzootic in Europe during the late 1960s [14]. The subtype VIb from pigeons with its likely origin from Middle East was responsible for the third panzootic during the 1980s [15]. The VII and VIII genotypes resulted in recent panzootics in Far East, Europe and South Africa since 1980s [16], [17]. Of the known genotypes, the viruses that originated before 1960s have a genome length of 15,186-nt and are also termed early genotypes, while the recent genotypes (originated after 1960s) possess a genome length of 15,192-nt [10]. Residue substitutions G124S and K192N in the F gene of the ancient genotypes III and IV viruses resulted in the evolution of genotype I viruses, which further became genotype II viruses by additional L69M and D82E substitutions in the same gene [18].

Antigenic analysis of Indian NDV isolates with monoclonal antibodies indicated that there are unusual antigenic types in circulation India extending support for vaccine failures [19], [20]. However, the molecular characteristics of circulating NDV strains in India are largely unknown. Earlier reports on different NDV isolates from India have not attempted to genotype the viruses [19], [20], [21]. However, circulation of multiple genotypes of NDV in India has been recently reported based on the FPCS [22]. In this study, we performed genotypic and pathotypic characterization of two isolates of NDV recovered 13 years apart from two different avian species (chicken and pigeon) to infer the nature of circulating genotypes in India. Our results indicate that there is a continuous persistence of genotype IV strains in India.

## Materials and Methods

### Virus strains and pathogenicity

The NDV2 and NDV-2K3 viruses were isolated from a pooled homogenate of spleen and brain of a chicken in the year 1987 and a pigeon in the year 2000, respectively. The chicken isolate was from a commercial layer flock experiencing diarrhoea and lethargy. Petechial haemorrhages in the proventriculus and ulcers in the intestine were observed in affected birds. The pigeon was a free-range bird found dull and lethargic with neurological signs. The viruses were isolated and purified by the limiting dilution method in 10-day-old specific-pathogen-free (SPF) embryonated chicken eggs (ECE) using standard procedures [23]. The virus identity was confirmed by haemagglutination-inhibition (HI) assay with polyclonal chicken antiserum to NDV and also by reverse transcription-polymerase chain reaction (RT-PCR) of the FPCS sequence [24]. The mean death time (MDT), intracerebral pathogenicity index (ICPI) and the ability to agglutinate chicken and equine erythrocytes were determined using standard procedures devised to distinguish these viruses [25], [26].

### RNA isolation and whole genome sequencing

RNA was extracted from two different pools of allantoic fluid; the cDNA was synthesized with superscript III RT mix (Invitrogen, CA) and sequencing of the complete genome of NDV-2K3 and NDV2 was performed with thirty six primer pairs similar to those reported in an earlier study [27] using high fidelity Taq DNA polymerase supermix (Invitrogen, CA). Some of the primer sequences were modified by inosine substitution based on the consensus sequence obtained by multiple sequence alignment of published NDV sequences (GenBank: EU293194, DQ060053, AY845500 and AF309418) (Table S1). The leader (3′end) and trailer (5′end) regions were generated by rapid amplification of cDNA ends (RACE) procedure, as described [28]. Sequencing of the different fragments was achieved with Big-Dye terminator v 3.1 kit (Applied biosystems, USA) for atleast three clones with both the forward and reverse primers to ensure a consensus and repeatable sequence data.

### Sequence analysis

The complete genome sequences of NDV-2K3 and NDV2 were obtained using the SeqMan module of the Lasergene V 7.1 (DNASTAR Inc, WI). Multiple alignment of the complete genome, the 374 bp sequence surrounding the FPCS and the M gene with corresponding sequences from GenBank were performed using ClustalW in MEGA 5.0 [29]. The GenBank accession number, country of origin and the genotype of NDV used in this study are described in Table S2. Following multiple alignment, the Bayesian Information Criterion (BIC), maximum likelihood values and Akaike Information Criterion corrected (AICc) scores were also determined for the maximum likelihood fits based on the data specific model to generate the phylogenetic tree and also to determine the genotype. The percentage identity and diversity for the respective coding regions, whole genome, leader and trailer were estimated using the MegAlign module of Lasergene V 7.1.

## Results

### Pathogenicity

The NDV-2K3 and NDV2 strains had a MDT of 40 and 46 hrs, while the ICPI values in chickens were 1.98 and 1.62, respectively, confirming the velogenic nature of these two viruses. The NDV-2 strain agglutinated only chicken but not equine erythrocytes while NDV-2K3 strain from pigeons agglutinated both chicken and equine erythrocytes.

### Sequence characteristics of the whole genome and phylogenetic analysis

The accession numbers for the complete genome sequences of NDV-2K3 and NDV2 are FJ986192 and GU187941, respectively. The genome lengths were 15,186 nucleotides (nt) similar to the reported length of most NDV strains from ‘early’ genotypes (1930–1960). The complete genome of these two strains also exhibited a very low divergence (4.1 to 7.2%) with the classical genotype IV strains than with the genotype II vaccine strains such as LaSota used in India. The length of the 3′ leader and 5′ trailer were 55 and 114 nt, respectively as reported for most NDV strains. The 5′ trailer also revealed a high level of conservation with the genotype IV strains (89.5 to 92.6%) while the level of divergence was higher with the vaccine strains such as Lasota, B1 and Mukteswar.

The gene order of 3′- N-P-M-F-HN-L-5′ coding from six open reading frames was similar to other APMV-1 strains. Comparisons of the different proteins with corresponding NDV strains with significant similarity and with common vaccine strains are shown in Table 1. The Indian strains had greater identity with the cognate proteins of well-characterized genotype IV NDV strains namely, Herts'33 and Italien than with the commonly used vaccine strains (Table 1). The divergence percentage across different proteins ranged from 0 to 8.9% with the N and L genes exhibiting higher similarity (96.4 to 100%) with genotype IV viruses.

**Table 1:** Features of the whole genome and the different protein sequences of NDV-2K3 and NDV2.

Regions compared		Gen-I(Ulster)AY562991		Gen-II(Lasota)AF077761		Gen-II(Bead-C)AF064091		Gen-III(Mukt)EF201805		Gen-IV(Herts/33)AY741404		Gen-IV(Italien)EU293914		Gen-V(USlargo)AY562990		Gen-VIIT/227-82AJ880277		Gen-VIIIndonesia 14698/80AY562985		Gen-VIIbPPMV-1 NY/1984FJ410145		Gen-VIIIQH4ChinaFJ751919		Gen-IXF48E8ChinaFJ436302		Gen-XIMG_1992HQ266603	
		FJ986192	GU187941	FJ986192	GU187941	FJ986192	GU187941	FJ986192	GU187941	FJ986192	GU187941	FJ986192	GU187941	FJ986192	GU187941	FJ986192	GU187941	FJ986192	GU187941	FJ986192	GU187941	FJ986192	GU187941	FJ986192	GU187941	FJ986192	GU187941
***Wh. Gene (2.9)***		13.2	11.8	16.6	15.4	16.6	15.4	12.4	11.0	**7.7**	**5.3**	**7.1**	**4.2**	13.2	11.9	14.1	13.0	15.4	14.3	14.5	13.4	14.6	13.3	12.7	11.2	14.6	12.7
***Leader (5.7)***		5.8	11.8	3.7	7.7	3.7	7.7	9.7	12.1	**11.8**	**5.8**	**11.8**	**5.8**	10.0	16.4	16.8	19.1	12.3	18.8	16.8	19.1	16.6	14.4	9.7	3.8	-	-
**Coding regions**	***N (0.4)***	6.2	6.4	9.6	9.8	9.6	9.8	6.0	6.2	**3.8**	**3.6**	**3.8**	**3.6**	4.9	5.1	4.6	4.9	6.2	6.4	4.9	5.1	5.3	5.5	5.3	5.5	6.2	6.4
	***P (0.3)***	13.9	13.9	15.8	15.8	15.8	15.8	13.6	13.6	**6.3**	**6.3**	**5.2**	**5.2**	16.1	16.1	17.3	17.3	17.0	17.0	17.7	17.7	17.0	17.0	13.6	13.6	17.3	17.3
	***M (1.4)***	14.2	13.2	16.2	15.5	16.2	15.5	12.2	11.6	**7.2**	**6.3**	**6.9**	**6.0**	10.0	9.0	10.3	9.3	12.2	11.2	11.2	10.3	12.8	11.9	13.2	11.9	11.2	10.3
	***F (1.1)***	8.8	9.2	10.5	10.5	10.5	10.5	8.4	8.0	**5.6**	**5.2**	**6.0**	**5.6**	11.3	10.9	9.0	8.6	8.4	8.0	9.2	8.8	9.6	9.0	8.2	7.4	13.0	12.6
	***HN (0.9)***	10.3	11.1	13.2	13.8	13.2	13.8	10.5	11.3	**8.1**	**8.9**	**7.5**	**8.3**	12.4	13.2	11.9	12.8	14.5	15.3	13.0	13.8	11.9	12.6	11.1	11.9	10.9	11.7
	***L (2.9)***	4.4	3.0	7.4	6.0	7.4	6.0	4.9	3.5	**3.2**	**0.5**	**2.9**	**0.0**	4.8	3.1	5.7	4.3	5.5	3.9	5.6	4.2	4.9	3.2	4.6	3.2	5.5	3.2
***Trailer (2.7)***		20.7	20.7	30.2	30.2	30.2	30.2	13.8	14.9	**9.5**	**10.5**	**7.4**	**6.5**	20.7	19.7	19.5	18.5	24.3	23.3	19.5	18.5	22.3	21.4	21.0	22.3	-	-

The Indian isolates FJ986192 and GU187941 have been compared with the sequences of standard strains (used in different publications) of Genotype I to XI.

***Note***:

• The whole genome comparison included from positions 67 to 15143 for strains of Genotype I to Genotype IV and 67 to 15148 for strains from Genotype IV (Herts/33) to Genotype IX as the leader and the trailer regions of Genotype XI (HQ266603) was not available.

• 6 nucleotide insertion in the 5′ UTR of Nucleoprotein of Genotypes V to XI and hence the genome size is 15192.

• The amino acids encoded by the matrix gene in all strains are 364 except for Herts /33 (380 aa). The amino acids encoded by the HN gene is 571 in all isolates except Lasota and Beaudette-C (577 aa) and Ulster (616 aa).

• The percentage divergence between Indian strains FJ986192 and GU187941 is shown within brackets. Percentage amino acid divergence is shown for the different coding regions. The lesser divergence in the different coding regions of FJ986192 and GU187941 with Herts/33 and Italien (bold faced) is shown.

• Whole genome and trailer region of FJ986192 and GU187941also reveals greater identity with Herts/33 and Italien than the other genotypes.

The deduced amino acid sequence of full-length F protein showed only a divergence of 5.2 to 6.0% with genotype IV strains, while the divergence ranged from 8 to 10.5% with currently used vaccine strains. The FPCS sequence had four basic amino acids ^112^ R-R-Q-R-R^116^ with a leucine (L) or a phenylalanine (F) residue at 117, respectively, in NDV-2K3 and NDV2. A C25W substitution compared to classical genotype IV strains was noticed while other cysteine residues were invariant in Indian genotype IV strains. The substitutions at positions T16I, V22T, A203T, T385A, K421R & M553I were unique to both Indian and the classical genotype IV viruses (Table 2). Both NDV-2K3 and NDV2 had unique substitutions at I121V, I269V, T288N, G358S, I516A, I520A, Y524H, K539R and T550A. In addition, NDV2 strain showed L117F, and T131P substitutions while NDV-2K3 showed and G124S substitution in the fusion peptide (Table S3). The L69M and D82E substitutions unique for genotype II viruses [18] were absent in the two Indian strains examined.

**Table 2:** Genotype specific amino acid substitution in the deduced Fusion protein sequences.

Genotype	Consensus amino acid and its position in the fusion protein																			
	11(V)	16(T)	22(E)	69(M)*	81(L)	82(E)*	121(I)*	192(K)*	269(I)	288(T)*	358(G)	385(T)	402(A)	479(D)	516(I)	520(V)	524(Y)	530(K)	550(T)	553(M)
**Genotype I**	-	-	-	-	-	-	-	-	-	-	-	-	-	-	-	-	-	-	-	-
**Genotype II**	A	-	V	**L**	-	**D**	-	-	-	-	-	-	V	N	-	I	-	-	-	-
**Genotype III**	-	-	A	-	-	-	-	N	-	-	-	-	-	-	-	-	-	-	-	-
**Genotype IV**	A^c^	I^b^	T^b^	-	-	-	***V*** c	N	V^c^	N^c^	S^c^	A^b^	V^c^	N^a^	A^c^V^a^	A^c^	H^c^	R^c^	A^c^	I^b^
**Genotype IX**	A	-	A	-	-	-	-	N	-	-	-	-	-	-	-	-	-	-	-	-
**Genotype XI**	I	-	A	-	V	-	-	-	-	-	-	-	-	N	V	-	-	-	-	I

*Genotype specific residue positions already reported (*Yu et al., 2001*).

a Residues at the indicated positions in classical genotype IV strains – Italian (EU293914) and Herts'33 (Ay741404).

b Residues at the indicated positions only in both the Indian and the classical genotype IV strains.

c Residues at the indicated positions found in genotype IV strains from India - NDV-2K3 (FJ986192) and NDV2 (GU187941) and not in the classical genotype IV virus.

• Eventhough the genotype XI strains groups in the same clade along with genotype IV there were several residues positions that were unique.

• Substitutions L69M and D82E reported for evolution of the genotype II (bold faced).

• The residue substitutions (V to A, A to V & D to N respectively) at positions 11, 402 & 479 respectively in Indian genotype IV strains are also observed in genotype II strains.

• Substitution V121I reported for the evolution of VIIb (bold italic).

With reference to HN, different amino acid sequence lengths of 571, 577, 581 and 616 has been reported for different NDV strains [30]. The predicted length of the HN protein of NDV-2K3 and NDV2 was 571 amino acids, which is a common feature of most virulent NDV strains [12], [30]. The Indian strains possessed a R516M substitution in the sialic acid binding site [31] and the neutralizing epitopes revealed D494N, V517D, S519G, S520Q and S521I substitutions compared to the classical genotype IV strains [32], [33], [34]. The other amino acid residues that have been identified to play different roles to the function of HN protein remain unchanged (Table S4).

**Figure 1:**
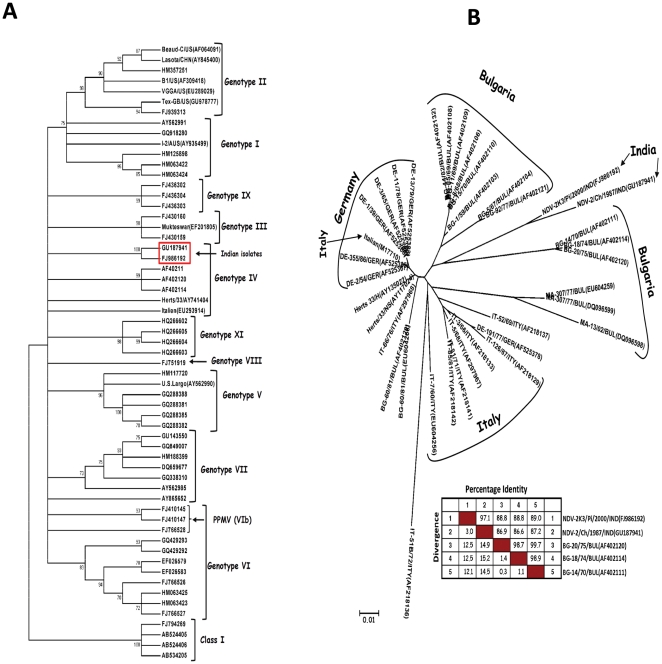
Phylogenetic relationship of Pigeon isolate NDV-2K3/Pi/2000/IND (FJ986192) and Chicken isolate NDV2/Ch/1987/IND (GU187941) from Tamil Nadu, India based on the 374-nucleotide sequence of the Fusion protein gene. **A:** A rooted consensus tree drawn to scale with 70% cut off value was obtained with the evolutionary history inferred by using the Maximum Likelihood method based a discrete gamma distribution model with evolutionary rate differences among sites, that also allowed some sites to be evolutionarily invariable (GTR+G+I) using MEGA5. *Note*: The NDV-2K3 (FJ986192) and NDV-2 (GU18794) from India (boxed) groups itself with Herts'33 (AY741404), Italian (EU293914) and other Bulgarian isolates in Genotype IV. The Class I APMV viruses (FJ794269, AB524405, AB524406 & AB534205) remain as an outgroup. **B:** An unrooted tree to show the relationship of 374 bp fusion protein gene of NDV-2K3 (FJ986192) and NDV-2 (GU187941) from Tamil Nadu, India with the other reported Genotype IV NDV strains from GenBank. *Note*: NDV-2K3 and NDV-2 from India groups with the Bulgarian genotype IV isolates (AF402111, AF402114 and AF402120). The percentage identity and divergence of the Indian isolates with these Bulgarian isolates are shown in the insert.

**Figure 2:**
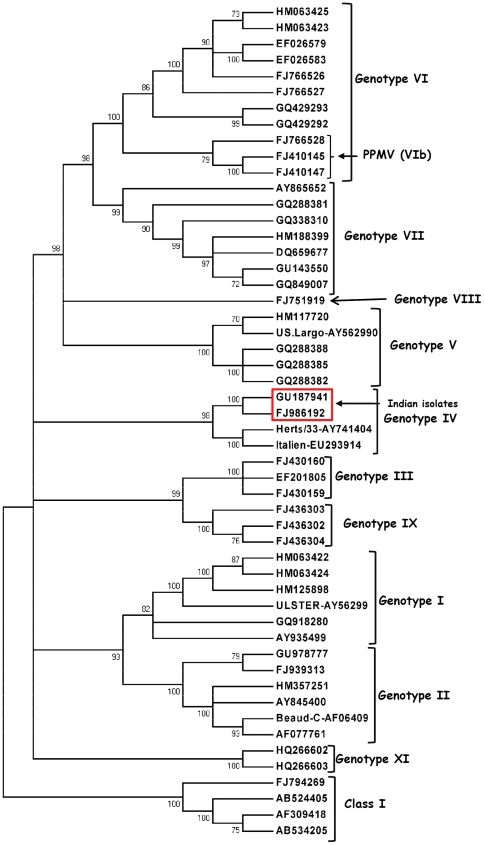
Phylogenetic analysis of the Matrix gene of the Pigeon isolate NDV-2K3/Pi/2000/IND (FJ986192) and Chicken isolate NDV-2/Ch/1987/IND (GU187941) Tamil Nadu, India. The assembly of the matrix sequences was performed using the Clustal W algorithm in MEGA 5. Sequences of previously published matrix sequences of NDV strains representing different genotypes have been included from the GenBank with their accession numbers. A rooted consensus tree drawn to scale with 70% cut off value was obtained with the evolutionary history inferred by using the Maximum Likelihood method based a discrete gamma distribution model with evolutionary rate differences among sites, that also allowed some sites to be evolutionarily invariable (GTR+G+I) using MEGA5. *Note*: The NDV-2K3 (FJ986192) and NDV-2 (GU18794) from India (boxed) groups itself with Herts'33 (AY741404), Italian (EU293914) even when the full-length matrix gene was utilized for the analysis. The Class I APMV viruses (FJ794269, AB524405, AB524406 & AB534205) remain as an outgroup.

**Figure 3:**
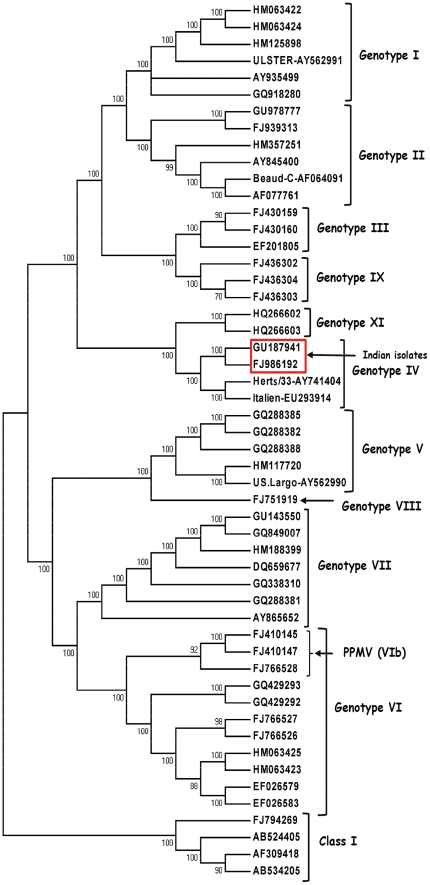
Phylogenetic analysis of the complete genome of Pigeon isolate NDV-2K3/Pi/2000/IND (FJ986192) and Chicken isolate NDV-2/Ch/1987/IND (GU187941) Tamil Nadu, India. The whole genome sequence of NDV strain 2K3/Chennai/Tamil Nadu (FJ986192) and NDV-2/Chicken/Namakkal/ Tamil Nadu (GU187941) was aligned with other NDV strain sequences from GenBank representing different genotypes using the Clustal W algorithm in MEGA 5.0. A rooted consensus tree drawn to scale with 70% cut off value was obtained with the evolutionary history inferred by using the Maximum Likelihood method based a discrete gamma distribution model with evolutionary rate differences among sites, that also allowed some sites to be evolutionarily invariable (GTR+G+I) using MEGA5. *Note*: The NDV-2K3 (FJ986192) and NDV-2 (GU18794) from India (boxed) groups itself with Herts'33 (AY741404), Italien (EU293914) even when the full-length genome was used for the analysis. The Class I APMV viruses (FJ794269, AB524405, AB524406 & AB534205) remained as outgroup.

A discrete gamma distribution model with evolutionary rate differences among sites, that also allowed some sites to be evolutionarily invariable (GTR+G+I) was found to be the best fit due to the lowest AICc score and BIC value for the phylogenetic analysis (374 bp F gene, matrix gene and the whole genome). The Indian strains grouped together with the ‘old’ genotype IV strains (Herts'33 and Italien or Bulgarian genotype IV strains) by phylogenetic analysis of the 374 bp of the F gene surrounding the FPCS (Figure 1). The topology of the phylogenetic tree and the grouping of the Indian strains remained the same when the full-length M gene and the whole genome sequences were compared with the sequences belonging to different genotypes from the GenBank (Figure 2 and Figure 3).

## Discussion

In this study, two NDV isolates recovered 13 years apart from a chicken and a pigeon from the southern part of India (Tamil Nadu) were genotypically and pathotyopically characterized. Both strains had a genome size of 15186 nt as that of the early NDV genotypes. Residue substitution analysis indicated that the Indian strains exhibited a very low divergence (0 to 8.9%) with genotype IV viruses than genotype II vaccine strains across the coding regions of different proteins. The Indian strains also clustered with genotype IV viruses (Herts'33 and Italien) when phylogenetic analysis was performed on 374 bp of the F gene or the full-length M gene or the complete genome, providing a strong evidence for the persistence of ‘genotype IV’ NDV strains in India. There have been no reports of this genotype to the GenBank database since 1989 from any part of the world [12], further confirming that the origin of this genotype in India is indigenous.

Most of the NDV strains isolated from pigeons do not result in significant disease in poultry and are considered mesogenic due to their ICPI values of 0.7 to 1.5 in chickens [35]–[36]. Also, typical pigeon isolates of virulent NDV (vNDV) producing neurological signs have been shown to be variants of genotype VIb with specific monoclonal antibody patterns and do not agglutinate chicken red blood cells (RBCs) [35] and hence classified as pigeon paramyxovirus-1 (PPMV-1) [36]. In this connection, the conserved M protein sequence analysis has been reliably used to differentiate the PPMV-1 and APMV-1 in earlier studies [37], [38], [39], [40]. Therefore, we have also used the phylogeny based on M gene sequence to classify and differentiate the nature of the NDV strain from pigeons. Analysis of the M gene sequence of NDV-2K3 isolate indicated its relatedness with Herts'33 and Italian isolate (AF124442 and EU293194) and agreed with an earlier report that NDV isolates from pigeons or doves also grouped with APMV-1 isolates from other species [39]. Also, the NDV2K3 strain agglutinated chicken and equine erythrocytes. Erythrocytes from chickens and humans contain only *N*-acetyl sialic acid (NeuAc) linked to galactose by alpha 2–3 and 2–6 linkages, whereas those from horses contain only *N*-glycolyl sialic acid (NeuGc) linked by alpha 2–3 linkages [41]. NDV strains from wild waterfowl agglutinated erythrocytes from all sources. By contrast, chicken isolates agglutinated erythrocytes from chicken and cow, but not those from horse and pig, with the exception of Ulster strain, which agglutinated pig erythrocytes [42], [43]. Direct analysis of receptor specificity of isolates from chicken and other aves using specific glycoconjugates is required for understanding these structural variations. The velogenic nature of the NDV-2K3 to chickens also suggests that NDV-2K3 is APMV-1 and not PPMV-1.

The molecular determinant of NDV pathogenicity in chickens is considered to be the FPCS sequence [24], [44], [45]. Velogenic and mesogenic strains possess a multibasic FPCS followed by phenylalanine at position 117 (R-X-K/R-R↓ F) [45]. Isolates lacking this motif have been shown to replicate only in the respiratory tract or gut of poultry and do not infect neural tissues [46]. However, recent analysis of the complete genome sequence of other APMV serotypes 2 through 9 and reverse genetic studies prove that the FPCS alone cannot confer virulence to an otherwise avirulent strain [47], [48]. NDV-2K3 and NDV2 had four basic amino acids ^112^ R-R-Q-R-R^116^ with leucine (L) or a phenylalanine (F) at position 117, respectively. Due to the absence of phenylalanine at 117 in the F protein of NDV-2K3, there might be other genome features such as the HN residues [49] that could have been responsible for the neurological signs in the pigeon noticed before death. Presence of a polybasic motif has been shown to increase the ICPI from 0.0 to 1.28. Along with these residues, the presence of arginine at 27 of F protein and cysteine at 123 of HN has also been shown to increase the ICPI to 1.5 [30]. A recent report also shows the mutation of the conserved 526 Tyrosine (Y) residue of HN reduced the neuraminidase, receptor binding and fusion activities as well attenuated the virulence of the virus to ECE and young birds [47] Thus, the presence of arginine (at 27 of F) and cysteine and tyrosine (in 123 and 526, respectively of HN) could also have contributed to the biological characteristics of the NDV-2K3 and NDV2 strains. Recent studies with L-chimeric viruses also have shown the contribution of L gene for the increased replication of the virus *in-vitro* and *in-vivo* [48]. The L protein amino acid sequence of NDV-2K3 and NDV2 also showed greater similarity (96.9 to 100%) to the Italian and Herts'33 strains, which could have been responsible for greater virulence of these isolates.

NDV genotypes II, III and IV have been reported to be responsible for the first panzootic of ND before the 1960s [16], with each genotype being restricted to a specific geographical area [50]. Genotype IV viruses, belonging to one of the ancient genotypes, were removed from the circulation in many parts of the world and the last reported genotype IV isolate was from Europe [10]. Intriguingly, genotype IV viruses were isolated in India in 1987 and 2000, suggesting that they are probably in circulation in India. The presence of this genotype in India was unknown earlier as there have been no efforts on genotyping of NDV, but others and we have recorded epizootic types of NDV in circulation based on monoclonal antibody typing [19], [20] and multiple genotypes based on the FPCS, recently [22]. The conventional vaccine strains used in India such as “Fuller” and “LaSota” belong to genotype II [12]. The other most commonly used vaccine strain in India the “Mukteswar”, which was reportedly derived from an Indian field isolate in 1940s, has been recently characterized as belonging to genotype III and is closely related to the Hertfordshire vaccine strain [51]. The two genotype IV Indian viruses had greater divergence to the commonly used vaccine strains such as LaSota. It has been recently reported that NDV vaccines formulated to be phylogenetically closer to the outbreak virus may provide a better ND control by reducing virus shedding and transmission from infected birds. There have been several reports suggesting that vaccination against NDV although protects against clinical disease, it fails to protect against virus shedding when challenged with heterologous genotypes [52], [53]. Therefore, it is quite possible conventional genotype II virus vaccines may not reduce transmission of genotype IV viruses in India allowing for continued circulation. Therefore, the risk of persistence or circulation of other genotypes than genotype II (LaSota and Fuller) and genotype III (Muketswar) vaccine strain is imminent. Further, the circulation of genotype IV viruses in free-range pigeons suggests the possibility of a reservoir in wild pigeons and possibly other aves [22] with periodic spillage into commercial chicken operations. From the results of our study, it could be concluded that more than one genotype of virulent NDVs including the ancient genotype IV viruses have been co-circulating in India.

### Ethics Statement

All animal work has been conducted in accordance with guidelines and approval of the Institutional animal ethical clearance committee of the Tamil Nadu Veterinary and Animal Sciences University, Chennai, India (approval# 1070/IAEC/AS2/DR/2006).

## Supporting Information

Table S1 **Oligonucleotide primers used to generate the overlapping amplicons encompassing the genome of NDV-2K3 ((FJ986192) and NDV2 (GU187941).**
(DOC)

Table S2 **Data on the NDV strains and sequences from the GenBank that has been used for analysis in this study.**
(DOC)

Table S3 **Comparison of amino acid substitutions in the important residues of the Fusion protein of NDV-2K3 (FJ986192) and NDV2 (GU187941) and other Vaccine Strains.**
(PDF)

Table S4 **Comparison of amino acid substitutions in the important residues of the HN protein of NDV-2K3 (FJ986192) and NDV2 (GU187941) and other Vaccine Strains.**
(PDF)

## References

[1] Mayo MA (2002b). A summary of taxonomic changes recently approved by ICTV.. Arch Virol.

[2] Chambers P, Millar NS, Bingham RW, Emmerson PT (1986). Molecular cloning of complementary DNA to Newcastle disease virus and nucleotide sequence analysis of the junction between the genes encoding the haemagglutinin-neuraminidase and the large protein.. J Gen Virol.

[3] Steward M, Vipond IB, Millar NS, Emmerson PT (1993). RNA editing in Newcastle disease virus.. J Gen Virol.

[4] Toyoda T, Sakaguchi T, Imai K, Inocencio NM, Gotoh B (1987). Structural comparison of the cleavage-activation site of the fusion glycoprotein between virulent and avirulent strains of Newcastle disease virus.. Virology.

[5] Glickman RL, Syddall RJ, Iorio RM, Sheehan JP, Bratt MA (1988). Quantitative basic residue requirements in the cleavage-activation site of the fusion glycoprotein as a determinant of virulence for Newcastle disease virus.. J Virol.

[6] Aldous EW, Mynn JK, Banks J, Alexander DJ (2003). A molecular epidemiological study of avian paramyxovirus type 1 (Newcastle disease virus) isolates by phylogenetic analysis of a partial nucleotide sequence of the fusion protein gene.. Avian Pathol.

[7] Kim LM, King DJ, Curry PE, Suarez DL, Swayne DE (2007a). Phylogenetic diversity among low-virulence Newcastle disease viruses from waterfowl and shorebirds and comparison of genotype distributions to those of poultry-origin isolates.. J Virol.

[8] Snoeck CJ, Ducatez MF, Owoade AA, Faleke OO, Alkali BR (2009). Newcastle disease virus in West Africa: new virulent strains identified in non-commercial farms.. Arch Virol.

[9] Maminiaina OF, Gil P, Briand FX, Albina E, Keita D (2010). Newcastle disease virus in Madagascar: identification of an original genotype possibly deriving from a died out ancestor of genotype IV.. PLoS One.

[10] Czegledi A, Ujvari D, Somogyi E, Wehmann E, Werner O (2006). Third genome size category of avian paramyxovirus serotype 1 (Newcastle disease virus) and evolutionary implications.. Virus Res.

[11] Kim LM, King DJ, Suarez DL, Wong CW, Afonso CL (2007b). Characterization of class I Newcastle disease virus isolates from Hong Kong live bird markets and detection using real-time reverse transcription-PCR.. J Clin Microbiol.

[12] Miller PJ, Decanini EL, Afonso CL (2010). Newcastle disease: Evolution of genotypes and the related diagnostic challenges.. Infect Genet Evol.

[13] Alexander DJ (2001). Gordon memorial lecture.. Newcastle disease Br Poult Sci.

[14] Alexander DJ, Saif YM, Barnes HJ, Glisson JR, Fadly AM, McDougald LR, Swayne DE (2003). Newcastle disease virus, other avian paramyxoviruses, and pneumovirus infections.. Disease of poultry, 11th ed.

[15] Kaleta EF, Alexander DJ, Russel PH (1985). The first isolation of the avian PMV-1 virus responsible for the current panzootic in pigeons?. Avian Pathol.

[16] Lomniczi B, Wehmann E, Herczeg J, Ballagi-Pordany A, Kaleta EF (1998). Newcastle disease outbreaks in recent years in Western Europe were caused by an old (VI) and a novel genotype (VII).. Archives of Virology.

[17] Liu XF, Wan HQ, Ni XX, Wu YT, Liu WB (2003). Pathotypical and genotypical characterization of strains of Newcastle disease virus isolated from outbreaks in chicken and goose flocks in some regions of China during 1985–2001.. Arch Virol.

[18] Yu L, Wang Z, Jiang Y, Chang L, Kwang J (2001). Charactrization of newly emerging Newcastle disease virus isolates from the people's republic of China and Taiwan..

[19] Kumanan K, Elankumaran S, Vijayarani K, Palaniswami KS, Padmanaban VD (1992). Characterization of Newcastle disease viruses isolated in India.. Zentralbl Veterinamed B.

[20] Roy P, Venuvopalan AT, Manvell R (2000). Characterization of Newcastle Disease Viruses Isolated from Chickens and Ducks in Tamilnadu, India.. Vet Res Comm.

[21] Kumanan K, Mathivanan B, Vijayarani K, Gandhi AA, Ramadass P (2005). Biological and molecular characterization of Indian isolates of Newcastle disease virus from pigeons.. Acta Virologica.

[22] Tirumurugaan KG, Vinupriya MK, Vijayarani K, Kumanan K (2011). Analysis of the Fusion Protein Cleavage Site of Newcastle disease virus Isolates from India Reveals Preliminary Evidence for the Existence of II, VI and VII Genotypes.. Indian J Virology.

[23] Alexander DJ, Purchase HG, Arp LH, Domermuth CH, Pearson JE (1989). Newcastle disease.. A laboratory manual for the isolation and identification of avian pathogens.

[24] Seal BS, King DJ, Bennett JD (1995). Characterization of Newcastle disease virus isolates by reverse transcription PCR coupled to direct nucleotide sequencing and development of sequence database for pathotype prediction and molecular epidemiological analysis.. J Clin Microbiol.

[25] Winslow NS, Hanson RP, Upton E, Brandly CA (1950). Agglutination of mammalian erythrocytes by Newcastle disease virus.. Proc Soc Exp Biol.

[26] Allan WH, Lancaster JE, Toth B (1978).

[27] Kattenbelt JA, Stevens MP, Gould AR (2006). Sequence variation in the Newcastle disease virus genome.. Virus Research.

[28] Li Z, Yu M, Zhang H, Wang HY, Wang LF (2005). Improved rapid amplification of cDNA ends (RACE) for mapping both the 5′ and 3′ terminal sequences of paramyxovirus genomes.. J Virol Meth.

[29] Tamura K, Peterson D, Peterson N, Stecher G, Nei M (2011). MEGA5: Molecular Evolutionary Genetics Analysis using Maximum Likelihood, Evolutionary Distance, and Maximum Parsimony Methods.. Mol Biol Evol.

[30] Romer-Oberdorfer A, Veits J, Werner O, Mettenleiter TC (2006). Enhancement of pathogenicity of Newcastle disease virus by alteration of specific amino acid residues in the surface glycoproteins F and HN.. Avian Diseases.

[31] Connaris H, Takimoto T, Russell R, Crennell S, Moustafa I (2002). Probing the Sialic Acid Binding Site of the Hemagglutinin-Neuraminidase of Newcastle Disease Virus: Identification of Key Amino Acids Involved in Cell Binding, Catalysis, and Fusion.. J Virol.

[32] Iorio RM, Glickman RL, Riel AM, Sheehan JP, Bratt MA (1989). Functional and neutralization profile of seven overlapping antigenic sites on the HN glycoprotein of Newcastle disease virus: monoclonal antibodies to some sites prevent viral attachment.. Virus Research.

[33] Iorio RM, Syddall RJ, Sheehan JP, Bratt MA, Glickman RL (1991). Neutralization map of the hemagglutinin-neuraminidase glycoprotein of Newcastledisease virus: domains recognized by monoclonal antibodies that prevent receptor recognition.. J Virol.

[34] Yusoff K, Nesbit M, Samson ACR, Emmerson PT (1988). Location of epitopes within the haemagglutinin-neuraminidase (HN) protein of newcastle disease virus by sequencing the HN genes of monoclonal antibody-resistant mutants.. Virus Research.

[35] Ujvari D, Wehmann E, Kaleta EF, Werner O, Savic V (2003). Phylogenetic analysis reveals extensive evolution of avian paramyxovirus type 1 strains of pigeons (Columba livia) and suggests multiple species transmission.. Virus Research.

[36] Alexander D, Parsons G (1984). Avian paramyxovirus type 1 infections of racing pigeons (2). Pathogenicity experiments in pigeons and chickens.. The Veterinary Record.

[37] Afzal MA, Pickford AR, Yates PJ, Forsey T, Minor PD (1994). Matrix protein gene sequence of vaccine and vaccine-associated strains of mumps virus.. J Gen Virol.

[38] Rota PA, Bloom AE, Vanchiere JA, Bellini WJ (1994). Evolution of the nucleoprotein and matrix genes of wild-type strains of measles virus isolated from recent epidemics.. Virology.

[39] Zanetti F, Rodriguez M, King DJ, Capua I, Carrillo E (2003). Matrix protein gene sequence analysis of avian paramyxovirus 1 isolates obtained from pigeons.. Virus Genes.

[40] Seal BS (1996). Analysis of matrix protein gene nucleotide sequence diversity among Newcastle disease virus isolates demonstrates that recent disease outbreaks are caused by viruses of psittacine origin.. Virus Genes.

[41] Collins MS, Bashiruddin JB, Alexander DJ (1993). Deduced amino acid sequences at the fusion protein cleavage site of Newcastle disease viruses showing variation in antigenicity and pathogenicity.. Arch Virol.

[42] Ito T, Suzuki Y, Mitnaul L, Vines A, Kida H (1997). Receptor specificity of influenza A viruses correlates with the agglutination of erythrocytes from different animal species.. Virology.

[43] Ito T, Kawaoka Y, Kameda C, Yasuda J, Kida H (1999). Differences in receptor specificity between Newcastle disease viruses originating from chickens and waterfowl.. J Vet Med Sci.

[44] Aldous EW, Alexander DJ (2001). Detection and differentiation of Newcastle disease virus (avian paramyxovirus type 1).. Avian Pathology.

[45] Pritzer E, Kuroda K, Garten W, Nagai Y, Klenk HD (1990). A host range mutant of Newcastle disease virus with an altered cleavage site for proteolytic activation of the F protein.. Virus Research.

[46] Hooper PT, Hanson E, Young JG, Russell GM, Della-Porta AJ (1999a). Lesions in the upper respiratory tract in chickens experimentally infected with Newcastle disease viruses isolated in Australia.. Aust Vet J.

[47] Khattar SK, Yan Y, Panda A, Collins PL, Samal SK (2009). A Y526Q mutation in the Newcastle disease virus HN protein reduces its functional activities and attenuates virus replication and pathogenicity.. J Virol.

[48] Rout SN, Samal SK (2008). The large polymerase protein is associated with the virulence of Newcastle disease virus.. J Virol.

[49] Huang Z, Panda A, Elankumaran S, Govindarajan D, Rockemann D (2004). The hemagglutinin neuraminidase protein of Newcastle disease virus determines tropism and virulence.. J Virol.

[50] Herczeg J, Wehmann E, Bragg RR, Travassos Dias PM, Hadjiev G (1999). Two novel genetic groups (VIIb and VIII) responsible for recent Newcastle disease outbreaks in Southern Africa, one (VIIb) of which reached Southern Europe.. Archives of Virology.

[51] Czegledi A, Wehman E, Lomniczi B (2003). On the origins and relationships of Newcastle disease virus vaccine strains Hertfordshire and Mukteswar, and virulent strain Herts'33.. Avian Pathology.

[52] Miller PJ, King DJ, Fonso CL, Saurez DL (2007). Antigenic differences among Newcastle disease virus strains of different genotypes used in vaccine formulation affect viral shedding after a virulent challenge.. Vaccine.

[53] Kapczynski DR, King DJ (2005). Protection of chickens against overt clinical disease and determination of viral shedding following vaccination with commercially available Newcastle disease virus vaccines upon challenge with highly virulent virus from the California 2002 exotic Newcastle disease outbreak.. Vaccine.

