# Supplementary material for: Genotypic and Pathotypic Characterization of Newcastle Disease Viruses from India
Source: PLoS One. 2011 Dec 9;6(12):e28414. doi: 10.1371/journal.pone.0028414 (PMC3235129; doi:10.1371/journal.pone.0028414)
Supplement: Table S1 — Oligonucleotide primers used to generate the overlapping amplicons encompassing the genome of NDV-2K3 ((FJ986192) and NDV2 (GU187941). (DOC) [file pone.0028414.s001.doc]

**Table S1: Oligonucleotide primers used to generate the overlapping amplicons encompassing the genome of NDV-2K3 ((FJ986192) and NDV2 (GU187941)**

| **Fragment Name** | **Primer Sequence**  **(5’ to 3’)** | **Product Size** |
| --- | --- | --- |
| **AF** | **^10^** ACC AAA CAG AGA ATC **I**GT **I**AG T **^31^** | 281bp |
| **AR** | **^291^** GAG GCA GAA TAC C**I**C AAA G**I**T CCA **^270^** |  |
| **BF** | **^208^** GTA CCT TAA AGG TAG A**I**G TCC C**I**G TAT T **^236^** | 454bp |
| **BR** | ****** |  |
| **CF** | **^615^** ATC CTC TCT ATC CAG GC**I** CAA GTA TGG **^642^** | 615bp |
| **CR** | ****** |  |
| **DF** | ****** |  |
| **DR** | ****** |  |
| **EF** | ****** | 501bp |
| **ER** | ****** |  |
| **PITF1F** | **^1868^** CCA GCG GAT TAG GGT GAA GA **^1887^** | 622bp |
| **PITF1R** | **^2490^** GGA GCG CAT GAG GGG TTA C **^2472^** |  |
| **GF** | ****** | 522bp |
| **GR** | ****** |  |
| **PB1F3F** | **^2541^** TTT GTG CAA GCG ATG ATG TC **^2560^** | 505bp |
| **PB1F3R** | **^3056^** CGC GCT TGA TTT TCC TGA T **^3039^** |  |
| **IF** | **^3412^** GAT CCA GCG CCT TGA CT**I** GTG G **^3434^** | 469bp |
| **IR** | **^3881^** ACC TTC AAT ACT GCA G**I**T GG **^3858^** |  |
| **JF** | **^3836^** AGG ATG TCT ACA AGA TCC C **^3858^** | 461bp |
| **JR** | **^4297^** G**T**T GGG **T**AC C**I**G CTT GGA TAA T **^4272^** |  |
| **KF** | ****** | 647bp |
| **KR** | ****** |  |
| **LF** | ****** | 639bp |
| **LR** | **^4850^** TAT CCT ACG GAT AGA **I**TC ACC AAG **I**GG **^4824^** |  |
| **MF** | ****** | 606bp |
| **MR** | ****** |  |
| **NF** | **^5226^** CCT TAA **ACA** AGC TGA CTA T **^5248^** | 597bp |
| **NR** | **^5849^** AAA GTT ATC CCG **I**CT AAG GAT AA **^5823^** |  |
| **OF** | ****** | 711bp |
| **OR** | **^6435^** GGC TAA C**I**G CGC GGT CCA T **^6413^** |  |
| **QF** | ****** | 597 bp |
| **QR** | **^7543^** ATA GAT AAG AT**A** GCC TGC TG **^7520^** |  |
| **HNF** | **^6412^** A TGG ACA GCG CAG TTA GCC AAG **^6433^** | 1650bp |
| **RR** | **^8071^** AG**A** GTA TT**I** GAT ATT TC**I** GCA ATG CT **^8042^** |  |
| **HNF** | **^6412^** A TGG ACA GCG CAG TTA GCC AAG **^6433^** | 2000bp |
| **SR** | **^8501^** GGT AGC CCA GTT AAT TTC CA **^8478^** |  |
| **TF** | ****** | 578bp |
| **TR** | **^8987^** TCA CCA ATT TGT TGG C**I**G C **^8964^** |  |
| **UF** | ****** | 755bp |
| **UR** | ****** |  |
| **VF** | **^9483^** TTG C**I**G CAA A**I**G CAG T**I**A GGA G **^9508^** | 234bp |
| **VR** | **^9747^** TC**A** AAT TCA AG**I** GCA GAT AA **^9720^** |  |
| **WF** | **^9576^** AAC GG**I** TAC AGA AAG AA **^9601^** | 708bp |
| **WR** | **^10384^** GCC CAT CA**I** CTG ATT GAT GGC **^10359^** |  |
| **XF** | **^10305^** AAA **AGT** CTG TCT TAA TTG G **^10328^** | 792bp |
| **XR** | **^11097^** AAA GA**I** ATG TCC TCA ATC CA **^11073^** |  |
| **YF** | **^11025^** GTT **I**TC CAT CAC **I**AA CA**I** **I**TC **^11050^** | 794bp |
| **YR** | **^11819^** GTT CTA T**C**G TAT CAG GAT TAG A **^11793^** |  |
| **ZF** | ****** | 1077bp |
| **B2F** | ****** | 715bp |
| **B2R** | ****** |  |
| **C2F** | **^13363^** AGG GAA AG**I** GAG GAC AG **^13384^** | 613bp |
| **C2R** | ****** |  |
| **D2F** | **^13903^** AGA GTT CCG TCC A**I**T ATG G **^13924^** | 1479bp |
| **E2R** | ****** |  |

****** Primer sequence information as reported in earlier publication [*Kattenbelt et al., 2006*]

The sequence information of the Leader and Trailer regions were generated and confirmed by 5’ and 3’ RACE respectively
