# Supplementary material for: Genotypic and Pathotypic Characterization of Newcastle Disease Viruses from India
Source: PLoS One. 2011 Dec 9;6(12):e28414. doi: 10.1371/journal.pone.0028414 (PMC3235129; doi:10.1371/journal.pone.0028414)
Supplement: Table S2 — Data on the NDV strains and sequences from the GenBank that has been used for analysis in this study. (DOC) [file pone.0028414.s002.doc]

Table S2: Data on the NDV strains and sequences from the GenBank used for analysis

| **S.No** | **Strain/Origin** | **Host** | **Accession Number** | | **Genotype** |
| --- | --- | --- | --- | --- | --- |
|  | Beaudette C | *Mesogenic strain* | AF064091 | Genotype II | |
|  | LaSota, CHINA | *Vaccine strain* | AY845400 | Genotype II | |
|  | NDV-4 (Tamil Nadu, India) I | *Chicken* | HM357251 | Genotype II | |
|  | Strain B1, USA | *Vaccine strain* | AF309418 | Genotype II | |
|  | VG/GA, USA |  | EU289029 | Genotype II | |
|  | TeX/GB/1948, USA | *Chicken* | GU978777 | Genotype II | |
|  | NDV/Chicken/Egypt/1/2005, Egypt | *Chicken* | FJ939313 | Genotype II | |
|  | Lasota | *Vaccine strain* | AF077761 | Genotype II | |
|  | Strain R8, China: Guang-dong | *Rallus aquaticus* | HM063424 | Genotype I | |
|  | Strain D3, Guang-dong , China | *Feral migratory ducks* | HM063422 | Genotype I | |
|  | WDK/JX/7793/2004, China: Jiangxi | *Duck* | HM125898 | Genotype I | |
|  | Strain I-2, Australia | *Chicken* | AY935499 | Genotype I | |
|  | BHG/Sweden/94, Sweden. | *Black-headed gull* | GQ918280 | Genotype I | |
|  | Ulster/67, United Kingdom: N. Ireland. | *Chicken* | AY562991 | Genotype I | |
|  | F48E8, China | *NA* | FJ436302 | Genotype IX | |
|  | FJ/1/85/Ch, China | *Chicken* | FJ436304 | Genotype IX | |
|  | ZJ/1/86/Ch, China | *Chicken* | FJ436303 | Genotype IX | |
|  | JS/9/05/Go, China | *Goose* | FJ430160 | Genotype III | |
|  | Mukteswar, Lanzhou, Chin | *Chicken* | EF201805 | Genotype III | |
|  | JS/7/05/Ch, China | *Chicken* | FJ430159 | Genotype III | |
|  | NDV-2 (Tamil Nadu, India) | *Chicken* | GU187941 | Genotype IV | |
|  | NDV-2K3 (Tamil Nadu, India) | *Pigeon* | FJ986192 | Genotype IV | |
|  | Herts’33 | *NA* | AY741404 | Genotype IV | |
|  | Italien | *NA* | EU293914 | Genotype IV | |
|  | MA-307/77, Bulgaria | *NA* | DQ096599 | Genotype IV | |
|  | MA-13/02, Bulgaria | *NA* | DE096598 | Genotype IV | |
|  | DE-355/86, Germany | *NA* | AF525385 | Genotype IV | |
|  | DE-13/79, Germany | *NA* | AF525383 | Genotype IV | |
|  | DE-11/78, Germany | *NA* | AF525380 | Genotype IV | |
|  | DE-191/77, Germany | *NA* | AF525378 | Genotype IV | |
|  | DE-3/65, Germany | *NA* | AF525368 | Genotype IV | |
|  | DE-2/54, Germany | *NA* | AF525367 | Genotype IV | |
|  | DE-1/39, Germany | *NA* | AF525366 | Genotype IV | |
|  | BG-44/82, Bulgaria | *NA* | AF402132 | Genotype IV | |
|  | BG-60/81, Bulgaria | *NA* | AF402129 | Genotype IV | |
|  | BG-92/77, Bulgaria | *NA* | AF402121 | Genotype IV | |
|  | BG-20/75, Bulgaria | *NA* | AF402120 | Genotype IV | |
|  | BG-18/74, Bulgaria | *NA* | AF402114 | Genotype IV | |
|  | BG-14/70, Bulgaria | *NA* | AF402111 | Genotype IV | |
|  | BG-15/70, Bulgaria | *NA* | AF402110 | Genotype IV | |
|  | BG-11/69, Bulgaria | *NA* | AF402109 | Genotype IV | |
|  | BE-85/69, Bulgaria | *NA* | AF402108 | Genotype IV | |
|  | BG-8/68, Bulgaria | *NA* | AF402106 | Genotype IV | |
|  | BG-5/67, Bulgaria | *NA* | AF402104 | Genotype IV | |
|  | BG-1/59, Bulgaria | *NA* | AF402103 | Genotype IV | |
|  | BG-60/81, Bulgaria | *NA* | EU604266 | Genotype IV | |
|  | MA-307/77, Bulgaria | *Chicken* | EU604259 | Genotype IV | |
|  | IT-7/60, Italy | *Chicken* | EU604256 | Genotype IV | |
|  | IT-52/69, Italy | *NA* | AF525378 | Genotype IV | |
|  | IT-126/87, Italy | *NA* | AF218129 | Genotype IV | |
|  | IT-3/66, Italy | *NA* | AF218133 | Genotype IV | |
|  | IT-5/68, Italy | *NA* | AF297967 | Genotype IV | |
|  | IT-81/71, Italy | *NA* | AF218141 | Genotype IV | |
|  | IT-85/81, Italy | *NA* | AF218142 | Genotype IV | |
|  | IT-51B/72, Italy | *NA* | AF218136 | Genotype IV | |
|  | MG_725_08, Madagascar | *Chicken* | HQ266602 | Genotype XI | |
|  | MG_39_4_08, Madagascar | *Chicken* | HQ266605 | Genotype XI | |
|  | MG_MEOLA_08, Madagascar | *Chicken* | HQ266604 | Genotype XI | |
|  | MG_1992, Madagascar. | *Chicken* | HQ266603 | Genotype XI | |
|  | QH4, China | *NA* | FJ751919 | Genotype VIII | |
|  | NDV-P05, Puebla , Mexico | *Chicken* | HM117720 | Genotype V | |
|  | U.S./Largo/71, USA | *NA* | AY562990 | Genotype V | |
|  | Cormorant/US(CA)/92-23071/1997 | *Cormorant* | GQ288388 | Genotype V | |
|  | Cormorant/US(CA)/D9704285/1997 | *Cormorant* | GQ288381 | Genotype V | |
|  | Cormorant/Canada/98CNN3-V1125/1998 | *Cormorant* | GQ288382 | Genotype V | |
|  | Cormorant/US(WI)/18719-03(USGS)/2003 | *Double crested Cormorant* | GQ288385 | Genotype V | |
|  | Go/CH/HLJ/LL01/08, China | *Goose* | GU143550 | Genotype VII | |
|  | JSD0812, China | *Laying Duck* | GQ849007 | Genotype VII | |
|  | SDWF02, China | *Ducklings and Duck embryos* | HM188399 | Genotype VII | |
|  | NA-1, China | *NA* | DQ659677 | Genotype VII | |
|  | ND/03/044, China | *NA* | GQ338310 | Genotype VII | |
|  | Cockatoo/Indonesia/14698/90, Indonesia | *Cockatoo* | AY562985 | Genotype VII | |
|  | Sterna/Astr/2755/2001, Russia | *Sterna albifrons pallas* | AY865652 | Genotype VII | |
|  | PPMV-1/New York/1984, USA | *Pigeon* | FJ410145 | Genotype VIb | |
|  | PPMV-1/Maryland/1984, USA | *Pigeon* | FJ410147 | Genotype VIb | |
|  | NDV05-029, China | *Pigeon* | FJ766528 | Genotype VIb | |
|  | Dove/Italy/2736/00, Italy | *Dove* | GQ429293 | Genotype VI | |
|  | AV324/96, Ireland | *Pigeon* | GQ429292 | Genotype VI | |
|  | 0.025/Pigeon/Belgium/248VB/1998 | *Pigeon* | EF026579 | Genotype VI | |
|  | 1.3/Pigeon/Belgium/248VB/1998, Belgium. | *Pigeon* | EF026583 | Genotype VI | |
|  | JS/07/22/Pi, China | *Pigeon* | FJ766526 | Genotype VI | |
|  | P4, China: Guang-dong | *Wild pigeon* | HM063425 | Genotype VI | |
|  | W4, China: Guang-dong | *White-breasted water hen* | HM063423 | Genotype VI | |
|  | JS/07/16/Pi, China | *Pigeon* | FJ766527 | Genotype VI | |
|  | NDV08-004, China | *Duck* | FJ794269 | Class I | |
|  | Goose/Alaska/415/91 | *Goose* | AB524405 | Class I | |
|  | B1 | *Chicken* | AF309418 | Class I | |
|  | 9a3b | *NA* | AB534205 | Class I | |

*NA- Information not available*
