# Supplementary material for: Genotypic and Pathotypic Characterization of Newcastle Disease Viruses from India
Source: PLoS One. 2011 Dec 9;6(12):e28414. doi: 10.1371/journal.pone.0028414 (PMC3235129; doi:10.1371/journal.pone.0028414)
Supplement: Table S4 — Comparison of amino acid substitutions in the important residues of the HN protein of NDV-2K3 (FJ986192) and NDV2 (GU187941) and other Vaccine Strains. (PDF) [file pone.0028414.s004.pdf]

Table S4: Comparison of amino acid substitutions in the important residues of the HN protein of NDV-2K3 (FJ986192) and NDV2 (GU187941) and other Vaccine Strains

Lento	B1/AF309418	M D R A V S Q V A L E N D E R E A K N T W R L I F R I A I L F L T V V T L A I S V A S L L Y S M G A S T P S D L V G I P	[60]
Lento	LASOTA/AF077761	. . . .	
Meso	Beaud-C/AF064091	. . . .	
Meso	MUKTES/EF201805	. . S . . . . R . . . . D . . . . V . . . . A . L . M . I . . . V . A V A . A . . . E . . . . G . . . S . .	
Velo	Italien/EU293914	. . . . . G R . . . . . E . . . . F V . . . . . F L . I . I . . . . . A . A . V . . . E . . . S G	
Velo	Herts/33/AY741404	. . . . . . R . . . . . E . . . . F V . . . . . . L . I . I . . . . . A . A . V . . . E . . . . G	
Velo	NDV-2K3/FJ986192	. . . . . . K . . . . . E . . . . F V . . . V . I L . I . I . . . . . A . A . A . . K E . . . . D	
Velo	NDV2/GU187941	. . . . . . K . . . . . E . . . . F V . . . V . I L . I . I . . . . . A . A . A . . K E . . . . D	
Lento	B1/AF309418	T R I S R A E E K I T S T L G S N Q D V V D R I Y K Q V A L E S P L A L L N T E T T I M N A I T S L S Y Q I N G A A N N	[120]
Lento	LASOTA/AF077761	. . . .	
Meso	Beaud-C/AF064091	. . . .	
Meso	MUKTES/EF201805	. A . Y . . . . R . . . A . . . . S I . . . . T . .	
Velo	Italien/EU293914	. V . . . . A . S . . . . S V	
Velo	Herts/33/AY741404	. V . . . . A . S . . . . S V	
Velo	NDV-2K3/FJ986192	. V . . . . . A R . . . A . S . . . . S V	
Velo	NDV2/GU187941	. V . . . . . A R . . . A . S . . . . S V	
		174 175	
Lento	B1/AF309418	S G W G A P I H D P D Y I G G I G K E L I V D D A S D V T S F Y P S A F Q E H L N F I P A P T T G S G C T R I P S F D M	[180]
Lento	LASOTA/AF077761	. . . .	
Meso	Beaud-C/AF064091	. . . .	
Meso	MUKTES/EF201805	. . C . . . V . . . . T	
Velo	Italien/EU293914	. . C . . . V I	
Velo	Herts/33/AY741404	. . C . . . V I	
Velo	NDV-2K3/FJ986192	. . C . . . V . . . . T . . T I	
Velo	NDV2/GU187941	. . C . . . V . . . . T . . T I	
Lento	B1/AF309418	S A T H Y C Y T H N V I L S G C R D H S H S Y Q Y L A L G V L R T S A T G R V F F S T L R S I N L D D T Q N R K S C S V	[240]
Lento	LASOTA/AF077761	. . . .	
Meso	Beaud-C/AF064091	. . . .	
Meso	MUKTES/EF201805	. . . . H . . . . A	
Velo	Italien/EU293914	. . . . H . . . . N	
Velo	Herts/33/AY741404	. . . . H . . . . I . . . N	
Velo	NDV-2K3/FJ986192	. . . . Y . . . . N . . . . N	
Velo	NDV2/GU187941	. . . . Y . . . . . P . . . . H N . . . . . . G .	
		258 299	
Lento	B1/AF309418	S A T P L G C D M L C S K A T E T E E E D Y N S A V P T R M V H G R L G F D G Q Y H E K D L D V T T L F G D W V A N Y P	[300]
Lento	LASOTA/AF077761	. . . . V	
Meso	Beaud-C/AF064091	. . . . V	
Meso	MUKTES/EF201805	. . . . I . . . . K . V I . . S . . . . R	
Velo	Italien/EU293914	. . . . I . . . . S . V T . . S . . . . I . . . K	
Velo	Herts/33/AY741404	. . . . I . . . . S . V T . . S . . . . I . . . K	
Velo	NDV-2K3/FJ986192	. . . . V . . . . V I . . S . . . . E . . . . I . . . K	
Velo	NDV2/GU187941	. . . . F . . . . V . . . . V I . . S . . . . E . . . . I . . . K	

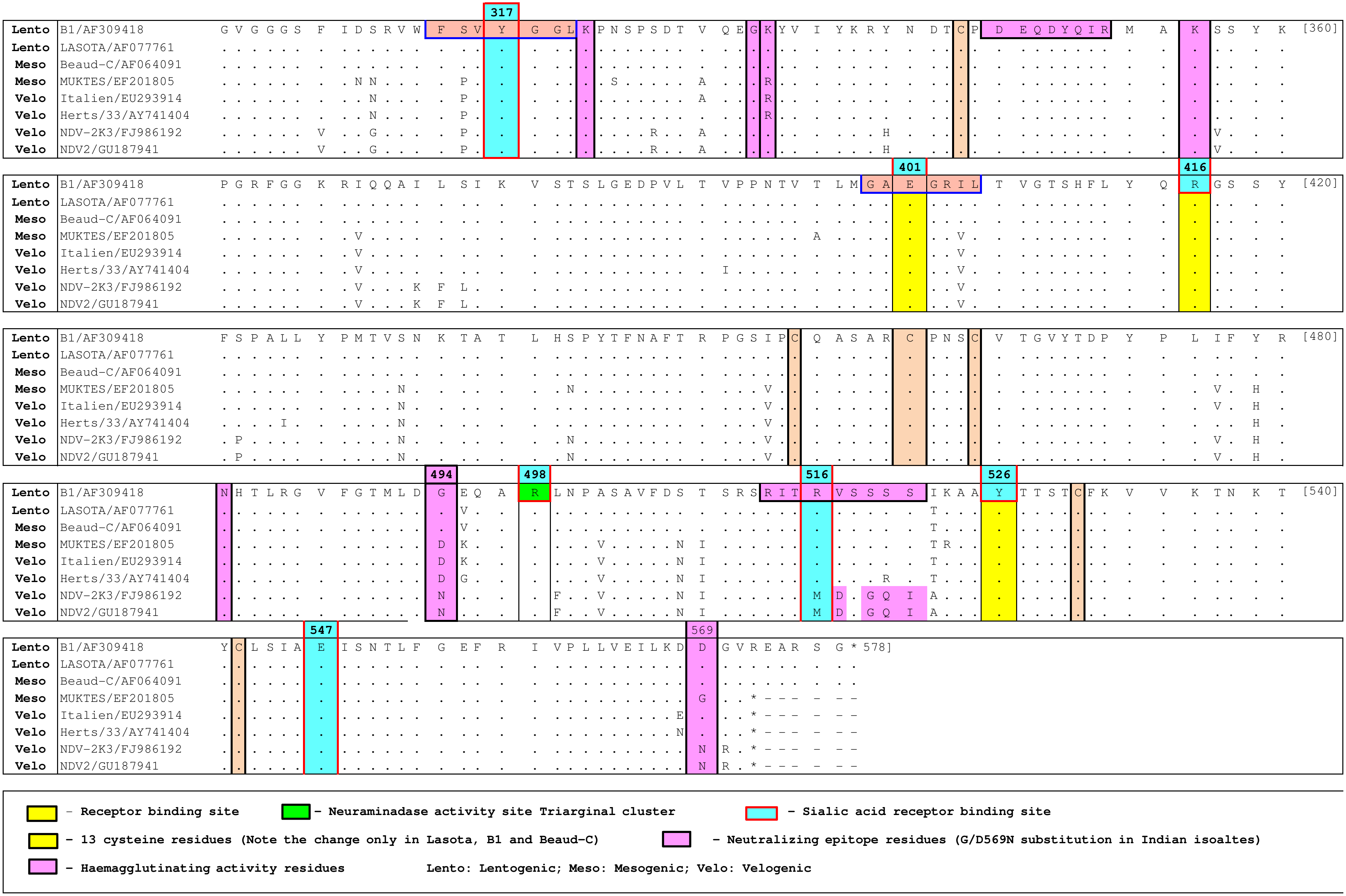
317
Lento B1/AF309418 GVGGGSFIDSRVWFSVYGGLKPNSPSDTVQEGKYVIYKRYNDTCPDEQDYQIRMAKSSYK [360]
Lento LASOTA/AF077761 ....
Meso Beaud-C/AF064091 ....
Meso MUKTES/EF201805 ........NN....P.......S.....A...R...........................
Velo Italien/EU293914 .........N....P.............A...R...........................
Velo Herts/33/AY741404 .........N....P.................R...........................
Velo NDV-2K3/FJ986192 ......V..G....P..........R..A..........H................V...
Velo NDV2/GU187941 ......V..G....P..........R..A..........H................V...
401
416
Lento B1/AF309418 PGRFGGKRIQQAILSIKVSTSLGEDPVLTVPPNTVTLMGAEGRILTVGTSHFLYQRGSSY [420]
Lento LASOTA/AF077761 ....
Meso Beaud-C/AF064091 ....
Meso MUKTES/EF201805 ........V..........................A.......V................
Velo Italien/EU293914 ........V..................................V................
Velo Herts/33/AY741404 ........V....................I.............V................
Velo NDV-2K3/FJ986192 ........V...KFL............................V................
Velo NDV2/GU187941 ........V...KFL............................V................
Lento B1/AF309418 FSPALLYPMTVSNKTATLHSPYTFNAFTRPGSIPCQASARCPNSCVTGVYTDPYPLIFYR [480]
Lento LASOTA/AF077761 ....
Meso Beaud-C/AF064091 ....
Meso MUKTES/EF201805 ...........N.......N............V.......................V.H.
Velo Italien/EU293914 ...........N....................V.......................V.H.
Velo Herts/33/AY741404 ....I......N....................V.........................H.
Velo NDV-2K3/FJ986192 .P.........N.......N............V.......................V.H.
Velo NDV2/GU187941 .P.........N.......N............V.......................V.H.
494
498
516
526
Lento B1/AF309418 NHTLRGVFGTMLDGEQARLNPASAVFDSTSRSRITRVSSSSIKAAYTTSTCFKVVKTNKT [540]
Lento LASOTA/AF077761 ..............V..........................T..................
Meso Beaud-C/AF064091 ..............V..........................T..................
Meso MUKTES/EF201805 .............DK......V.....NI............TR.................
Velo Italien/EU293914 .............DK......V.....NI............T..................
Velo Herts/33/AY741404 .............DG......V.....NI..........R.T..................
Velo NDV-2K3/FJ986192 .............N....F..V.....NI......MD.GQIA..................
Velo NDV2/GU187941 .............N....F..V.....NI......MD.GQIA..................
547
569
Lento B1/AF309418 YCLSIAEISNTLFGEFRIVPLLVEILKDDGVREARSG* 578]
Lento LASOTA/AF077761 ....
Meso Beaud-C/AF064091 ....
Meso MUKTES/EF201805 ....G..*------
Velo Italien/EU293914 ....E...*------
Velo Herts/33/AY741404 ....N...*------
Velo NDV-2K3/FJ986192 ....NR.*------
Velo NDV2/GU187941 ....NR.*------
- Receptor binding site
- Neuraminadase activity site Triarginal cluster
- Sialic acid receptor binding site
- 13 cysteine residues (Note the change only in Lasota, B1 and Beaud-C)
- Neutralizing epitope residues (G/D569N substitution in Indian isoaltes)
- Haemagglutinating activity residues
Lento: Lentogenic; Meso: Mesogenic; Velo: Velogenic
